# October

**Published:** 1884-10

**Authors:** 


### OCTOBER.

This is the month in which most of the colleges of the country open their doors after the summer vacation. From this time until April, what with professional engagements and regular lectures, college professors have a busy time of it. It is not the lectures alone that demand so much of their time, but every teacher whose heart is in his work (and if it is not he is wofully out of place), must be a constant student that he may keep fully abreast the advance of thought, and be prepared to place intelligently before students all the latest discovered remedies and the newest appliances. A competent teacher in any advancing profession has no leisure for rest. Should he for a moment attempt to rely upon a well-earned reputation and remain content with present acquirements, he will have the mortification to see the professional world moving past him, and will soon find himself in the rear instead of the vanguard, which is his proper place, while his pupils will seek a more progressive master. As colleges multiply, the strife for a leading position becomes more intense, and hence every college professor is constantly placed upon his mettle. This is a fact that should be taken into consideration by those who believe that we already have too many schools, and that what is needed is rather a better support for those already in existence.

It is a momentous question for a student to decide, when he is called upon to choose his Alma Mater. It is not alone the character, intelligence, and professional standing of the professors, but as every man has his individual characteristics, so every school is distinguished by peculiar features. The character of his probable class associates and the general tone which prevails at any specified school, should largely influence the student in making his choice. The relation of the school and its professors to the profession as a whole, and the question whether it is engaged in sustaining a wholesome professional code and placing its students upon high moral ground, are matters of great import. The school that is engaged in insidiously undermining the moral basis upon which a profession rests, that teaches, or connives at, or ignores questions of professional ethics, should be carefully avoided. We have in mind as illustrative of this a prominent college, one of whose professors in a lecture before the graduating class instructed them in the different ways by which the provisions of the wholesome state laws for the regulation of dental practice might be evaded and infringed. He absolutely gave them lessons in quackery, did what he could to place the students in antagonism to the reputable part of the profession, and lent his influence toward launching them upon professional life to wage a war upon their own flag, and make of them professional pirates at the outset. It need not be said that schools in which the moral tone is at so low an ebb should be shunned by students.

It is but natural that as a journalist we should feel the deepest interest in those institutions that are represented in our advertising pages, for our attention has by that presentation been specially called to them. We do not think that they are the only schools worthy of patronage, but it is certain that all of them are representatives of their class.

The Medical Department of the University of Buffalo has already commenced its thirty-ninth annual course of lectures. It was founded by men who have become eminent as teachers, practitioners and authors—Austin Flint, John C. Dalton, Frank H. Hamilton, and James P. White—and the high reputation that the college secured at the outset has ever been maintained. The didactic teaching of this school is not inferior to that of any college in America. Although it confers no diplomas in dentistry, it offers through special lectures by its corps of professors, as well as by the establishment of a chair for oral diseases, unusual inducements for dental students who desire to take their first, or first and second courses in a medical college.

Of the New York College of Dentistry it is unnecessary to speak at length. It has been too long and too favorably known to the dental profession to need extended notice. It is sufficient to say that never in its history was it so well prepared to offer every advantage to the student as it is to-day. The college is open during the entire year, and when there are no lectures the student is afforded opportunities for actual practice in the Infirmary, under the direction of capable demonstrators.

The Chicago College of Dental Surgery is a new institution, but it seems destined to take an important position. It is the only dental school in a great city, and therefore possesses all the facilities which such a city can offer. It takes high professional ground at the outset, and proposes to graduate no student who is not thoroughly qualified. From the high character, sound scholarship, and eminent professional attainments of its professors, it is not at all a hazardous matter to assure students that they will have every opportunity for securing thorough training and an intimate knowledge of all that is essential for them to know, and if after graduation they do not take high positions, the fault will be their own.

